# Time Kinetics and prognosis roles of calcitonin after surgery for medullary thyroid carcinoma

**DOI:** 10.1186/s12957-024-03397-3

**Published:** 2024-05-06

**Authors:** Fengli Guo, Guiming Fu, Fangxuan Li, Yitong Hua, Zhongyu Wang, Xiangqian Zheng, Jingzhu Zhao, Ming Gao

**Affiliations:** 1 Department of Thyroid and Neck Tumor, Key Laboratory of Cancer Prevention and Therapy, Tianjin Medical University Cancer Institute and Hospital, National Clinical Research Center for Cancer, Tianjin’s Clinical Research Center for Cancer, Tianjin, China; 2 Department of Thyroid and Breast Surgery, Binzhou Medical University Hospital, Binzhou, China; 3 Department of Thyroid-otolaryngology, Sichuan Cancer Center, School of Medicine, Sichuan Cancer Hospital & Institute, University of Electronic Science and Technology of China, Chengdu, China; 4 Department of cancer prevention, Tianjin Medical University Cancer Institute and Hospital, Tianjin, China; 5 Department of Thyroid and Breast Surgery, Tianjin Union Medical Center, Tianjin, China; 6 Tianjin Key Laboratory of General Surgery in construction, Tianjin Union Medical Center, Tianjin, China

**Keywords:** Medullary thyroid carcinoma, Calcitonin, Biochemical persistence, Biochemical recurrence, Structural recurrence

## Abstract

**Background:**

Medullary thyroid carcinoma (MTC) is a malignant tumor with low incidence. Currently, most studies have focused on the prognostic risk factors of MTC, whatever, time kinetic and risk factors related to calcitonin normalization (CN) and biochemical persistence/recurrence (BP) are yet to be elucidated.

**Methods:**

A retrospective study was conducted for 190 MTC patients. Risk factors related to calcitonin normalization (CN) and biochemical persistence/recurrence (BP) were analyzed. The predictors of calcitonin normalization time (CNT) and biochemical persistent/recurrent time (BPT) were identified. Further, the prognostic roles of CNT and BPT were also demonstrated.

**Results:**

The 5- and 10-year DFS were 86.7% and 70.2%, respectively. The 5- and 10-year OS were 97.6% and 78.8%, respectively. CN was achieved in 120 (63.2%) patients, whereas BP was presented in 76 (40.0%) patients at the last follow up. After curative surgery, 39 (32.5%) and 106 (88.3%) patients achieved CN within 1 week and 1 month. All patients who failed to achieve CN turned to BP over time and 32/70 of them developed structural recurrence. The median time of CNT and BPT was 1 month (1 day to 84 months) and 6 month (3 day to 63months), respectively. LNR > 0.23 and male gender were independent predictors for CN and BP. LNR > 0.23 (Hazard ratio (HR), 0.24; 95% CI,0.13–0.46; *P* < 0.01) and male gender (HR, 0.65; 95% CI, 0.42–0.99; *P* = 0.045) were independent predictors for longer CNT. LNR > 0.23 (HR,5.10; 95% CI,2.15–12.11; *P* < 0.01) was still the strongest independent predictor followed by preoperative serum Ctn > 1400ng/L (HR,2.34; 95% CI,1.29–4.25; *P* = 0.005) for shorter BPT. In survival analysis, primary tumor size > 2 cm (HR, 5.81; 95% CI,2.20-15.38; *P* < 0.01), CNT > 1 month (HR, 5.69; 95% CI, 1.17–27.61; *P* = 0.031) and multifocality (HR, 3.10; 95% CI, 1.45–6.65; *P* = 0.004) were independent predictor of DFS.

**Conclusion:**

Early changes of Ctn after curative surgery can predict the long-term risks of biochemical and structural recurrence, which provide a useful real-time prognostic information. LNR significantly affect the time kinetic of biochemical prognosis. Tumor burden and CNT play a crucial role in MTC survival, the intensity of follow-up must be tailored accordingly.

## Background

Medullary thyroid carcinoma (MTC) is a malignancy subtype originating from C cells of the thyroid gland, characterized with secreting calcitonin (Ctn) and carcinoembryonic antigen (CEA). Despite its low prevalence, MTC demonstrates a aggressive clinical course and is susceptible to lymph node involvement and distant metastases. Ctn is a highly sensitive biochemical marker indicating residual, recurrence or metastasis long prior to tumor localization can be visualized by imaging [1]. The postoperative normalization of serum Ctn levels is associated with a favorable outcome. Ideally, the serum Ctn decreases nadir to normal levels after curative surgery. Nonetheless, it is common for patients presenting persistent or recurrent biochemical non-normalization. Little data of the time regarding how long it takes for the serum Ctn level to decrease its nadir or when will it start to rise, is available. Additionally, the risk factors predicting serum Ctn normalization and biochemical persistence/recurrence are not well characterized.

Previous studies have reported that the serum Ctn levels declined rapidly within hours [2] and to undetectable levels within the first few days postoperative [3–5]. A reduction in Ctn levels of less than 50% 30 min after thyroidectomy plus central neck lymph node dissection suggests the persistence of tumor tissue in MTC patients [2]. For patients underwent curative surgery, the Ctn levels were undetectable in 97% (41/42) of patients 1 month after surgery and 100% in 6 months after surgery. With a median duration of follow-up of 4.5 years, 5 patients had detectable values at final follow up, and only two cases had structural evidence of disease [6]. Short-term Ctn normalization can’t guarantee long-term biochemical cure and completely eradicate structural recurrence. Patients with excellent response to therapy might experience biochemical and structural recurrence in 15% and 4% patients with longer follow-up [7]. However, little information is available regarding the kinetic changes of postoperative serum Ctn persistent/recurrent time. Serum Ctn doubling-time has drawn attention in studies as a prognostic indicator [8], but the calculation of doubling time requires a long-term interval or even several years.

Therefore, the present study was conducted to establish the time frame of calcitonin normalization (CN) and biochemical persistence/recurrence (BP) in patients with MTC, and to explore the associated clinical and pathological factors. Furthermore, we identified the independent predictors of calcitonin normalization time (CNT), biochemical persistent/recurrent time (BRT) and disease-free survival (DFS). To our knowledge, this is the first study covering both the CNT and BPT and long-term prognosis.

## Materials and methods

### Patients selection

A total of 190 patients who were first diagnosed with MTC and underwent curative surgery in Tianjin medical university cancer institute and hospital between February 2015 and February 2020 was included in present study. The medical records of the patients were retrospectively reviewed and followed up in the years. All patients were performed with total thyroidectomy and central node dissection. Lymph node dissection in the lateral neck was performed according to the neck ultrasound preoperative and evidence intro-operative. Patients with preoperative Ctn records and continuous postoperative Ctn monitoring were included in the study. Patients with preoperative Ctn ≤ 2 ng/L, those without thyroid or neck dissection and those loss follow-up as well as pediatric patients, were excluded. Postoperative pathological stage was classified according to the 8th revision of the American Joint Committee of Cancer (AJCC) TNM classification. Data regarding demographics, epidemiological, clinical and pathological, as well as preoperative and postoperative laboratory values were retrieved from electronic medical records.

### Biochemical assays and definitions

Ctn was measured using Immulite 2000® Siemens with a sex dependent reference range (male < 2-8.5 ng/L, female < 2–5 ng/L) and a detection limits: <2.0 ng/L and > 2,000 ng/L. CN, as serum Ctn normalization, was defined as serum Ctn levels decreased to < 5 ng/L for male and < 8.5 ng/L for female. CNT was calculated since the time from the last surgery to the time when serum Ctn normalization achieved. For patients who failed to achieve CN and had persistent or elevated serum Ctn, the time was calculated since last surgery to last follow-up. BP, was defined as serum Ctn never decreased, or raised from nadir for patients who failed to achieve CN, and exceeded 5ng/L or 8.5 ng/L for patients who achieved CN. BPT was calculated since last surgery to the time of serum Ctn raised for patients who failed to achieve CN and exceed upper limitation for patients who achieved CN. For patients without BP, the time was calculated from last surgery to the last follow-up.

Lymph node metastases ratio (LNR) defined as the number of lymph node involvement divided by the total number of dissected.

## Follow-up

Follow-up was performed early after surgery. The postoperative serum Ctn levels were measured within 3 days and a week in partial patients and 1 month in all patients, and repeated every 1–3 months intervals according to the results. The end of the surveillance period for each patient was considered the date of last follow-up or structural recurrence. Patients who underwent the initial and second operations within 3 months for curative intent were considered to be a single sequence and the serum Ctn levels after last operation were taken into account.

This study was approved by the ethics committee of Tianjin Medical University Cancer Institute and Hospital (EK2022260).

### Statistical analysis

Data were analyzed using SPSS software (SPSS for Windows, version 22.0). Continuous data were presented as means and standard deviations or median values with ranges, and analyzes with non-parametric test and t test for differences between groups. Discrete variables were described with rated (%) and analyzed by *x*^2^ test for differences between groups. The Cut-off of preoperative serum Ctn level and the LNR were determined by Receiver Operating Characteristic (ROC) curve. Multivariate logistic regression models were constructed to analyze the independent factors contributing to CN and BP. Kaplan-Meier and log-rank tests were used to estimate the CN and BP rates. Multivariate COX regression models were conducted to identify the independent factors contributing to CNT and BPT. In all cases, a *P* value < 0.05 (double tail) was considered statistically significant.

## Results

### Characteristics of patients

The study included 108 females and 92 males. The median age was 52 years (range 19–74) with a median follow-up period of 67 months (range 18–127). Multifocality presented in 55 patients (28.9%). According to 8th AJCC TNM classification, there were 58 (30.5%) patients in stage I, 22 patients in stage II, 25 patients in stage III and 85 patients in stage IV. Structural recurrence occurred in 35 (18.4%) patients at the last follow up. The distant organs most commonly affected by systemic spread were, in descending order, lung, bone and liver.

**Fig. 1:**
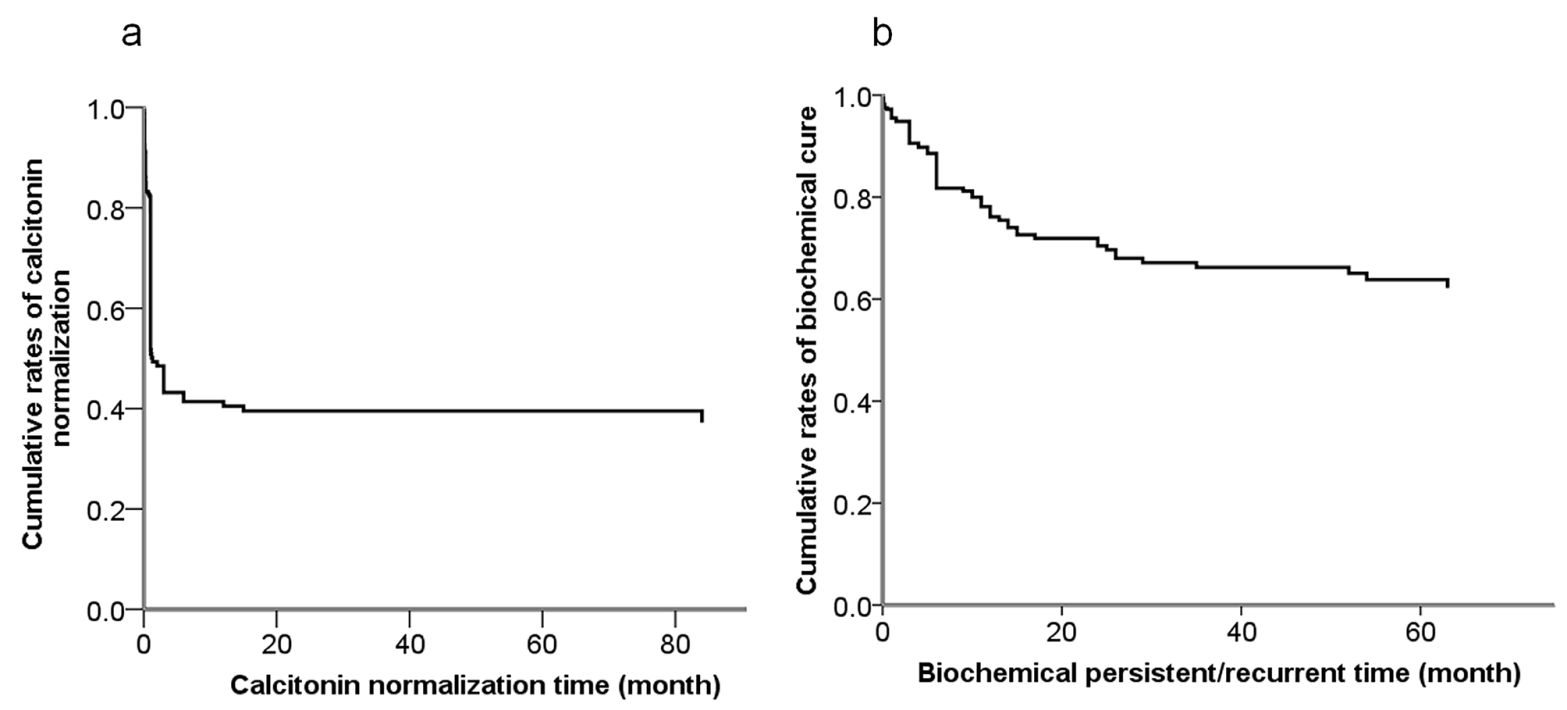
Cumulative rates of calcitonin normalization (CN) (**a**) and biochemical cure (**b**) of 190 MTC patients

At the last follow-up, 120 (63.2%) patients achieved CN, whereas 70 (36.8%) patients failed. For patients who achieved long term biochemical cure, the serum Ctn levels decreased sharply in several days after curative surgery. The time-dependent curve of cumulative rates of CN decreased rapidly, eventually leveling off more than 1 month, and 21 (17.5%), 18 (15.0%), 67 (55.8%), 14 (11.7%) patients achieved CN within 3 days, 1 week, 1 week to 1 month, more than 1 months after surgery, respectively. Only 6 patients had a slow decline in serum Ctn as long as more than 6 months or even more than a year, up to 84 months. The median time of CNT was 1 month with a range of 1d − 84 months. Of 120 patients, 95.0% (114) patients remained CN status with a median follow up time of 66.7 months, and only 3 patients demonstrated evidence of structural disease. The time-dependent curve of cumulative rates of CN and BP was shown in Fig. 1.

**Fig. 2:**
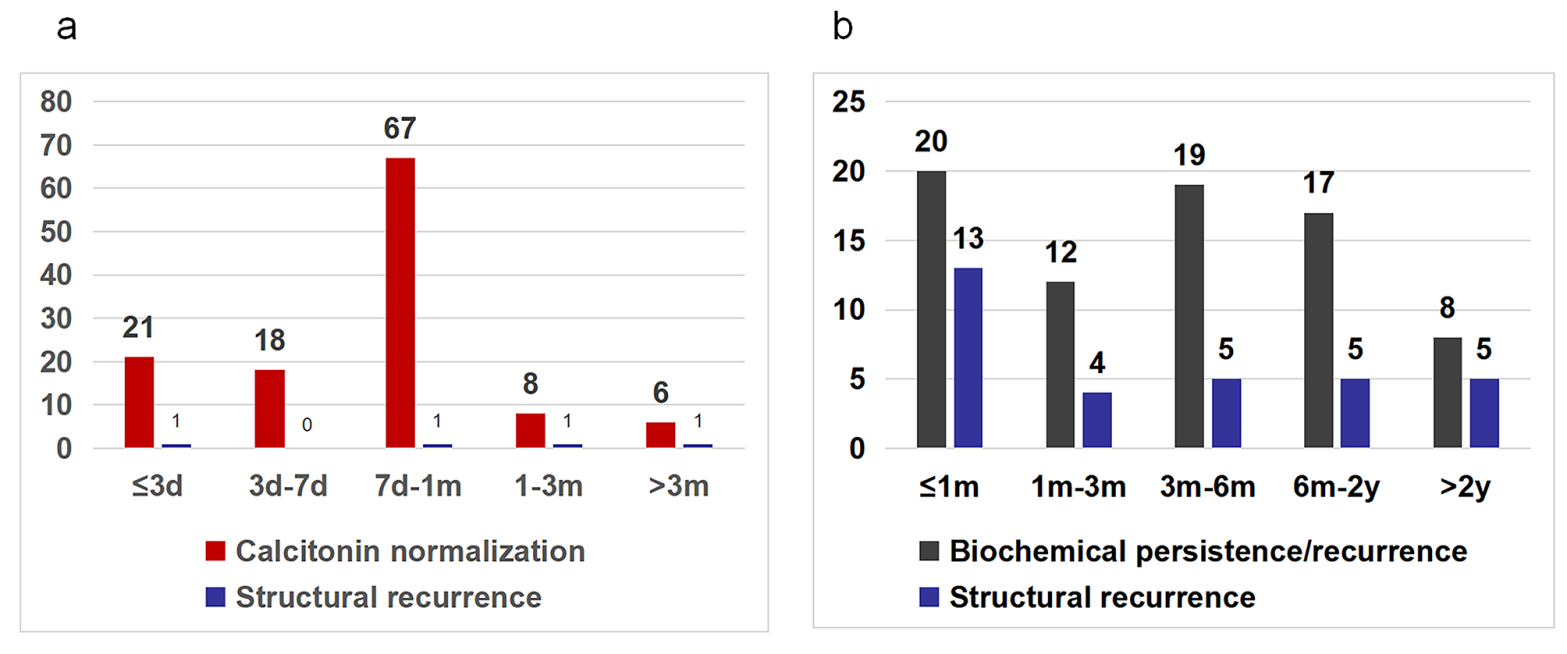
The distribution of calcitonin normalization time (CNT) (**a**) and biochemical persistent/recurrent time (BPT) (**b**) with structural recurrence. *d, day/days; m, month/months; y, year/years

There were 76 (40.0%) patients developed BP including 6 patients previously achieved CN. Postoperative Ctn decreased to varying degrees, and then rises from nadir at different points of time. Consequently, all patients who failed to achieve CN all progressed to BP over time. The median time of CNT was 6 months with a range of 3 days − 63 months. The time-dependent curve of cumulative rates of BP advanced steadily up to 2 years, eventually leveling off more than 2 years. There were 20 (26.3%), 12 (15.8%), 19 (25.0%), 17 (22.4%) and 8 (10.5%) patients developed recurrence within 1 month, 1 to 3 months, 3 to 6 months and 6 months to 2 years and longer than 2 years, with the longest time being 63 months. About 42.1% (32/76) patients with BP progressed to structural recurrence at the last follow-up and 65.0% (13/20) patients recurrent within 1 month, including 8 patients nearly without decrease in Ctn presenting structural recurrence after short-term follow-up. Nearly half of(32/70)patients failed to achieve CN progressed to structural recurrence. The distributions of CNT and BPT with structural recurrence were presented in Fig. 2.

### Comparison of clinical, pathological and biological indicators based on CN and BP

To calculate the cutoff values of preoperative serum Ctn levels and LNR for the CN and BP, the patients were grouped into CN (*n* = 120) and failure (*n* = 70), BP (*n* = 76) and biochemical cure (*n* = 114), respectively.

**Fig. 3:**
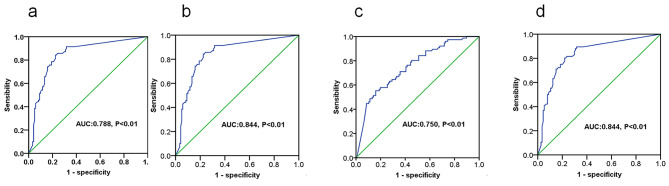
Identification of the optimal cutoff values of preoperative Ctn and LNR based on CN and BP via ROC curve analysis. The optimal cutoff values of preoperative Ctn (**a**) and LNR (**b**) based on CN were 0.23 and 1400 pg/L, respectively. The optimal cutoff values for preoperative serum Ctn (**c**) and LNR (**d**) based on BP were 0.23 and 1400 pg/L, respectively

The cut-off value of preoperative Ctn levels and LNR were identified by ROC cure analysis. When Youden index was the largest, the preoperative serum Ctn level and LNR were both 1400ng/L and 0.23 based on CN and BP, respectively. Accordingly, the cut-off value of preoperative Ctn level and LNR was set as 1400ng/L and 0.23, respectively (Fig. 3).

The clinicopathological factors associated with CN and BP were analyzed. Male gender, multifocality, extrathyroid invasion, primary tumor size > 2 cm, advanced T stage (T3/T4) and N stage (N1a/N1b), advanced clinical stage, LNR > 0.23, preoperative serum Ctn > 1400ng/L were all related to CN and BP significantly (*P* < 0.05). Table 1 listed clinical, pathological and biological factors related to CN and BP.

**Table 1:** The clinical, pathological and biological factors associated with Calcitonin normalization (CN) and Biochemical recurrence (BR).

Characteristic	CN				BR			
	Yes (*n*, %)	No (*n*,%)	x^2^	*P*	No (*n*, %)	Yes (*n*,%)	x^2^	*P*
Gender			18.71	< 0.01			18.87	< 0.01
Female	80 (76.9)	24 (23.1)			77 (74.0)	27 (26.0)		
Male	40 (46.5)	46 (53.5)			37 (46.0)	49 (57.0)		
Age (year)			0.28	0.596			0.75	0.385
≤ 50	76 (61.8)	47 (38.2)			71 (57.7)	52 (42.3)		
> 50	44 (65.7)	23 (34.3)			43 (64.2)	24 (35.8)		
Multifocality			17.84	< 0.01			15.35	< 0.01
Solitary	98 (72.6)	37 (27.4)			93 (68.9)	42 (31.1)		
Multiple	22 (40.0)	33 (60.0)			21 (38.2)	34 (61.8)		
Extrathyroid invasion			23.56	< 0.01			20.76	< 0.01
No	101 (73.7)	36 (26.3)			96 (70.1)	41 (29.9)		
Yes	19 (35.8)	34 (64.2)			18 (34.0)	35 (66.0)		
Primary tumor size (cm)			17.57	< 0.01			12.05	< 0.01
≤ 2	91 (74.0)	32 (26.0)			85 (69.1)	38 (30.9)		
> 2	29 (43.3)	38 (56.7)			27 (43.3)	38 (56.7)		
T stage			19.31	< 0.01			14.68	< 0.01
T1/T2	104(71.7)	41 (28.3)			98 (67.6)	47 (32.4)		
T3/T4	16 (35.6)	29 (64.4)			16 (35.6)	29 (64.4)		
N stage			55.85	< 0.01			56.63	< 0.01
0	74 (90.2)	8 (9.8)			72 (87.8)	10 (12.2)		
N1a	19 (67.9)	9 (32.1)			18 (64.3)	10 (35.7)		
N1b	27 (33.8)	53 (66.2)			24 (30.0)	56 (70.0)		
Clinical stage			46.87	< 0.01			47.59	< 0.01
I/II	73 (91.3)	7 (8.8)			71 (88.8)	9 (11.3)		
III/IV	47 (42.7)	63 (57.3)			43 (39.1)	67 (60.9)		
LNR			68.12	< 0.01			62.63	< 0.01
≤ 0.23	93 (89.4)	11 (10.6)			89 (85.6)	15 (14.4)		
> 0.23	27 (31.4)	59 (65.9)			25 (29.1)	61 (70.9)		
Preoperative serum Ctn(ng/L)			36.98	< 0.01			29.30	< 0.01
≤ 1,400	97 (75.8)	31 (24.2)			91 (71.1)	37 (28.9)		
> 1,400	15 (27.8)	39 (72.2)			15 (27.8)	39 (72.2)		

^*^P < 0.05,CN, calcitonin normalization; BR, biochemical recurrence; Ctn, calcitonin; LNR, lymph node metastasis ratio

Logistic analysis was performed to analyze the independent factors for CN and BP. LNR > 0.23 (Odd ratio (OR),15.06; 95% Confidence interval (CI),4.27–53.09; *P* < 0.01) and male gender (OR, 2.67; 95% CI,1.08–6.58; *P* = 0.034) were independent predictors for CN. LNR > 0.23 (OR,9.78; 95% CI,3.20-29.92; *P* < 0.01) and male gender (OR, 2.52; 95% CI,1.09–5.82; *P =* 0.030) were independent predictors for BP. The results were listed in Table 2.

**Table 2:** Logistic analysis of the clinical, pathological and biological factors of Calcitonin normalization (CN) and Biochemical recurrence (BR).

Characteristic	CN			BR		
	OR	95% CI	*P* value	OR	95% CI	*P* value
Male	2.67	1.08–6.58	0.034	2.52	1.09–5.82	0.030
Multifocality	2.58	0.97–6.83	0.057	1.95	0.78–4.88	0.151
Extrathyroid invasion	3.18	0.95–10.71	0.062	2.68	0.87–8.23	0.086
Primary tumor size > 2 cm	2.02	0.64–6.39	0.231	1.43	0.48–4.24	0.519
T3/T4	1.16	0.29–4.68	0.835	1.03	0.27–3.86	0.970
N stage			0.182			0.095
N1a	0.28	0.01–7.82	0.454	0.31	0.02–6.53	0.452
N1b	0.79	0.03–21.79	0.890	1.02	0.05–20.54	0.992
III/IV stage	1.51	0.07–33.39	0.795	1.65	0.10-28.74	0.730
LNR > 0.23	15.06	4.27–53.09	< 0.01	9.78	3.20-29.92	< 0.01
Preoperative serum Ctn > 1,400 ng/L	2.77	0.91–8.48	0.073	2.24	0.76–6.58	0.144

^*^P < 0.05, CN, calcitonin normalization; BR, biochemical recurrence; Ctn, calcitonin; LNR, lymph node metastasis ratio; OR, odds ratio; CI, confidence interval

### Clinical, pathological and biological predictors of CNT and BPT

For patients who achieved CN and failed, male gender, multifocality, primary tumor size > 2 cm, advanced T stage (T3T/4 vs. T1/T2), N stage (N0 vs. N1a/1b, N1a vs. N1b) and clinical stage (III/IV vs. I/II), LNR > 0.23, preoperative serum Ctn > 1400ng/L were significant predictors for longer CNT (*P* < 0.05) in univariate analysis. In the adjusted multivariate analysis, LNR > 0.23 (HR, 0.24; 95% CI,0.13–0.46; *P* < 0.01) and male gender (HR, 0.65; 95% CI, 0.42–0.99; *P* = 0.045) were independent predictors for longer CNT. The results were listed in Table 3.

**Table 3:** Univariate and multivariate analysis of clinical, pathological and biological predictors of calcitonin normalization time (CNT).

Characteristic	Univariate Kaplan-Meier analysis			Multivariate COX analysis		
	¯x ± SD	x^2^	*P*	HR	HR 95.0% CI	*P*
Gender		20.81	< 0.01			
Female	27.35 ± 4.70					
Male	64.35 ± 6.31			0.65	0.42–0.99	0.045
Age (year)		0.18	0.669			
≤ 50	46.08 ± 5.16					
> 50	40.75 ± 6.63					
Multifocality		15.93	< 0.01			
Solitary	33.75 ± 4.51					
Multiple	69.80 ± 7.58			0.66	0.40–1.09	0.105
Extrathyroid invasion		19.55	< 0.01			
No	30.98 ± 4.32					
Yes	76.88 ± 7.23			0.66	0.36–1.23	0.191
Primary tumor size (cm)		15.98	< 0.01			
≤ 2	32.18 ± 4.65					
> 2	65.60 ± 4.91			0.84	0.50–1.44	0.530
T stage		15.20	< 0.01			
T1/T2	34.48 ± 4.40					
T3/T4	74.43 ± 8.14			0.91	0.47–1.76	0.775
N stage		64.22	< 0.01			
0	12.39 ± 3.87					0.053
N1a	39.89 ± 9.90			2.09	0.54–8.03	0.283
N1b	77.33 ± 6.01			0.95	0.26–3.48	0.936
Clinical stage		57.58	< 0.01			
I/II	11.21 ± 3.73					
III/IV	66.94 ± 5.38			0.90	0.27–3.02	0.858
LNR		71.17	< 0.01			
≤ 0.23	13.56 ± 3.56					
> 0.23	79.96 ± 5.70			0.24	0.13–0.46	< 0.01
Preoperative serum Ctn(ng/L)		35.43	< 0.01			
≤ 1,400	29.94 ± 4.46					
> 1,400	83.96 ± 7.00			0.54	0.29–1.03	0.063

*P < 0.05, CNT, calcitonin normalization time; Ctn, calcitonin; LNR, lymph node metastasis ratio; SD, standard deviation; HR, hazard ratio; CI, confidence interval

Since the patients who failed to achieve CN all progressed to BP, the biochemical failures and BP were almost identical, with the exception of 6 individuals switching from normalization to recurrence. Subsequently, all the predictors for longer CNT were predictors for BPT in univariate analysis. After multivariate COX analysis, LNR > 0.23 (HR,5.10; 95% CI,2.15–12.11; *P* < 0.01) was still the strongest independent predictors followed by preoperative serum Ctn > 1400ng/L (HR,2.34; 95% CI,1.29–4.25; *P* = 0.005) for shorter BPT. The results were listed in Table 4.

**Table 4:** Univariate and multivariate analysis of clinical, pathological and biological predictors to biochemical persistent/recurrent time (BRT).

Characteristic	Univariate Kaplan-Meier analysis			Multivariate COX analysis		
	‾x ± SD	x^2^	*P*	HR	HR 95.0% CI	*P*
Gender		20.24	< 0.01			
Female	95.68 ± 5.10					
Male	56.70 ± 6.17			1.58	0.93–2.67	0.088
Age (year)		0.91	0.341			
≤ 50	76.03 ± 5.32					
> 50	81.54 ± 6.68					
Multifocality		19.04	< 0.01			
Solitary	89.66 ± 4.72					
Multiple	41.65 ± 6.01			1.43	0.88–2.33	0.151
Extrathyroid invasion		24.31	< 0.01			
No	90.93 ± 4.65					
Yes	43.67 ± 7.08			1.37	0.78–2.40	0.280
Primary tumor size (cm)		16.04	< 0.01			
≤ 2	87.39 ± 4.71					
> 2	58.06 ± 7.32			1.09	0.56–2.13	0.800
T stage		21.02	< 0.01			
T1/T2	85.50 ± 4.12					
T3/T4	48.48 ± 8.67			1.17	0.61–2.25	0.638
N stage		63.49	< 0.01			
0	111.84 ± 4.32					0.072
N1a	75.07 ± 8.78			0.20	0.03–1.64	0.136
N1b	39.57 ± 5.57			0.44	0.06–3.24	0.419
Clinical stage		46.90	< 0.01			
I/II	113.09 ± 4.18					
III/IV	49.76 ± 5.02			3.29	0.43–24.96	0.250
LNR		72.37	< 0.01			
≤ 0.23	109.79 ± 3.98					
> 0.23	36.94 ± 5.05			5.10	2.15–12.11	< 0.01
Preoperative serum Ctn(ng/L)		47.70	< 0.01			
≤ 1,400	88.25 ± 4.37					
> 1,400	28.78 ± 5.34			2.34	1.29–4.25	0.005

*P < 0.05, BRT, biochemical persistent/recurrent time; Ctn, calcitonin; LNR, lymph node metastasis ratio; SD, standard deviation; HR, hazard ratio; CI, confidence interval

**Fig. 4:**
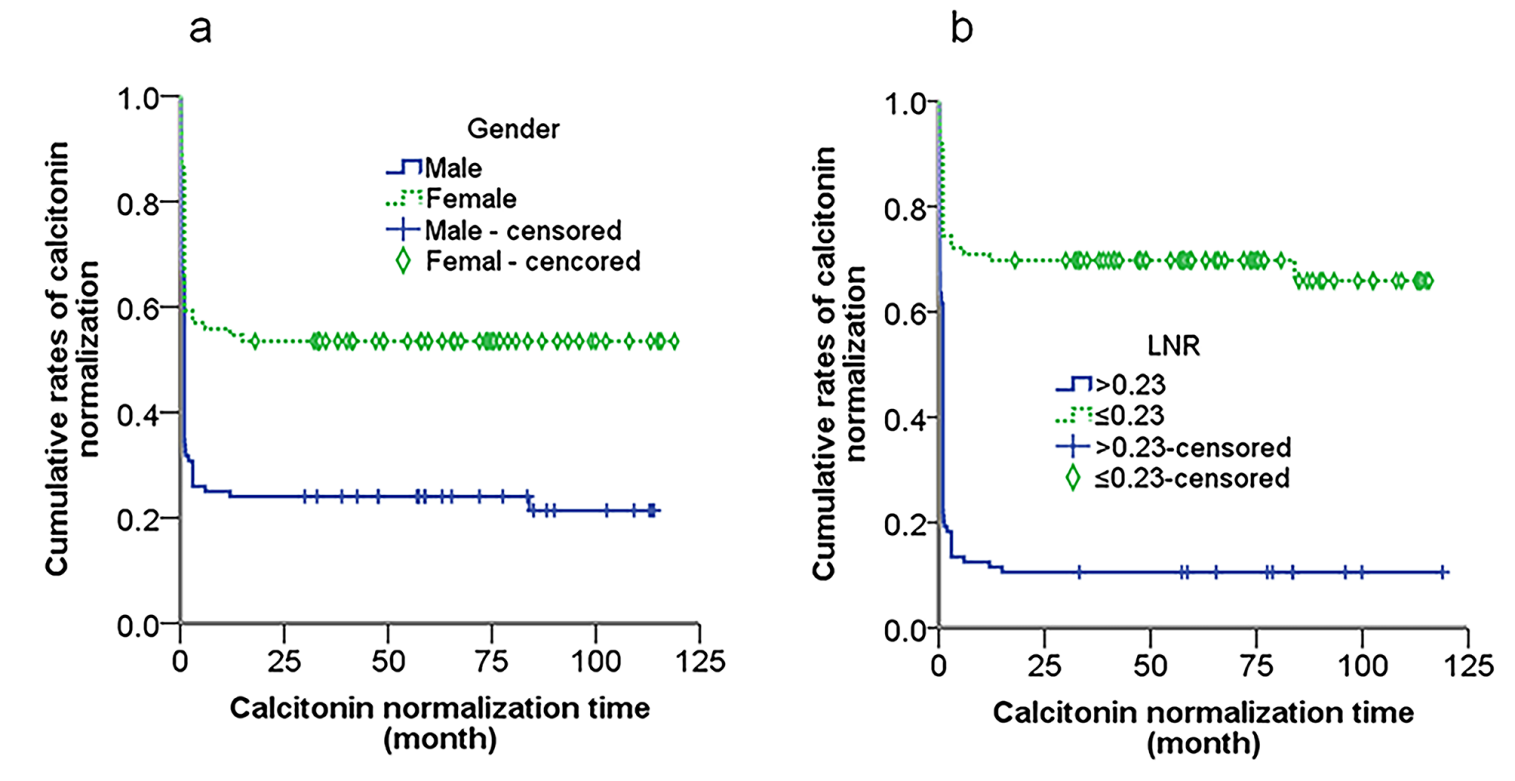
Time-dependent curve on CN of 190 MTC patients according to LNR cutoff and gender. Differences in CNT between groups were significant (*P*<0.05, log-rank test)

**Fig. 5:**
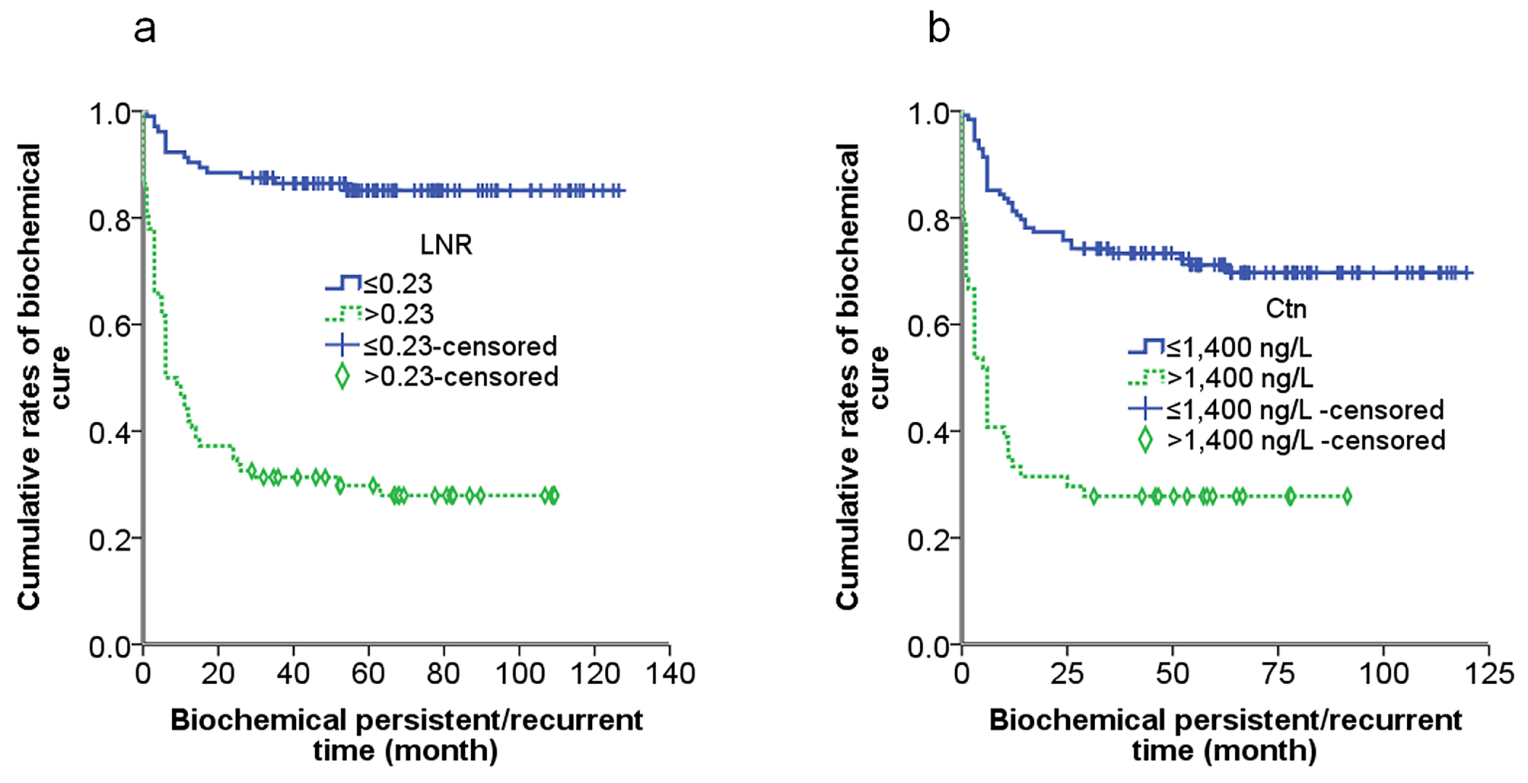
Time-dependent curve on BP of 190 MTC patients according to LNR cutoff (**a**) and preoperative serum Ctn cutoff (**b**). Differences in BPT between groups were significant (*P*<0.05, log-rank test)

Time-dependent curve on CN of 190 MTC patients according to gender and LNR cutoff were presented in Fig. 4. Time-dependent curves on BP of 190 MTC patients according to LNR and preoperative serum Ctn cutoff were presented in Fig. 5.

To compare the clinical and pathological characteristics of patients with different CNT and BPT, the 120 patients who achieved CN were divided into CNT ≤ 1 month (*n* = 103) and > 1 month (*n* = 17) group, and the 76 patients who developed BP were divided into BPT ≤ 3 month (*n* = 32) and > 3 month (*n* = 44) group, respectively. As demonstrated in Table 5, low N stage was related to CNT ≤ 1 month, primary tumor size ≤ 2 cm and preoperative serum Ctn ≤ 1,400 (ng/L) was related to BPT > 3 month (all *P* < 0.05), in subgroup analysis. Sixty-nine (93.2%) patients of N0 and 68 (93.2%) patients of I/II had shorter CNT (≤ 1 month). Table 5 demonstrated the clinical and pathological characteristics of MTC patients related to CNT ≤ 1 month or > 1 month, and BPT ≤ 3 month or > 3 month.

**Table 5:** Comparison of clinical and pathological characteristics of MTC patients with different calcitonin normalization time (CNT) and biochemical persistent/recurrent time (BRT).

Characteristic	CNT				BRT			
	≤ 1 month (*n*, %)	> 1 month (*n*,%)	x^2^/t/Z	*P*	≤ 3 month (*n*, %)	> 3 month (*n*,%)	x^2^/t/Z	*P*
Gender			0.14	0.711			1.32	0.250
Female	68 (85.0)	12 (15.0)			9 (33.3)	18 (66.7)		
Male	35 (87.5)	5 (12.5)			23 (46.9)	26 (53.1)		
Age (year)			0.92	0.337			3.04	0.081
≤ 50	67 (88.2)	9 (11.8)			25 (40.9)	26 (51.0)		
> 50	36 (81.8)	8 (18.2)			7 (28.0)	18 (72.0)		
Multifocality			0.57^#^	0.735			1.57	0.210
Solitary	83 (84.7)	15 (15.3)			15 (35.7)	27 (64.3)		
Multiple	20 (90.9)	2 (9.1)			17 (50.0)	17 (50.0)		
Extrathyroid invasion			0.88^#^	0.470			0.35	0.556
No	88 (87.1)	13 (12.9)			16 (39.0)	25 (61.0)		
Yes	15 (78.9)	4 (21.1)			16 (45.7)	19 (54.3)		
Primary tumor size (cm)			0.00^#^	1.000			5.40	0.020
≤ 2	78 (85.7)	13 (14.3)			11 (28.9)	27 (71.1)		
> 2	25 (86.2)	4 (13.8)			21 (55.3)	17 (44.7)		
T stage			0.32^#^	0.699			3.29	0.070
T1/T2	90 (86.5)	14 (13.5)			16 (34.0)	31 (66.0)		
T3/T4	13 (81.3)	3 (18.8)			16 (55.2)	13 (44.8)		
N stage			6.87^#^	0.032			3.46^#^	0.160
0	67 (90.5)	7 (9.5)			2 (20.0)	8 (80.0)		
N1a	17 (89.5)	2 (10.5)			3 (30.0)	7 (70.0)		
N1b	19 (70.4)	8 (29.6)			27 (48.2)	29 (51.8)		
Clinical stage			3.21	0.073			4.02^#^	0.071
I/II	66 (90.4)	7 (9.6)			1 (11.1)	8 (88.9)		
III/IV	37 (78.7)	10 (21.3)			31 (46.3)	36 (53.7)		
LNR			0.54^#^	0.532			3.75	0.053
≤ 0.23	81 (87.1)	12 (12.9)			3 (20.0)	12 (80.0)		
> 0.23	22 (81.5)	5 (18.5)			29 (47.5)	32 (52.5)		
Preoperative serum Ctn(ng/L)			2.63^#^	0.117			15.90	< 0.01
≤ 1,400	86 (88.7)	11 (11.3)			7 (18.9)	30 (81.1)		
> 1,400	11 (73.3)	4 (26.7)			25 (64.1)	14 (35.9)		

#Fisher exact test; *P < 0.05, CNT, calcitonin normalization time; BRT, biochemical persistent/recurrent time; Ctn, calcitonin; LNR, lymph node metastasis ratio

### Clinical, pathological and biological predictors of disease-free survival (DFS)

**Fig. 6:**
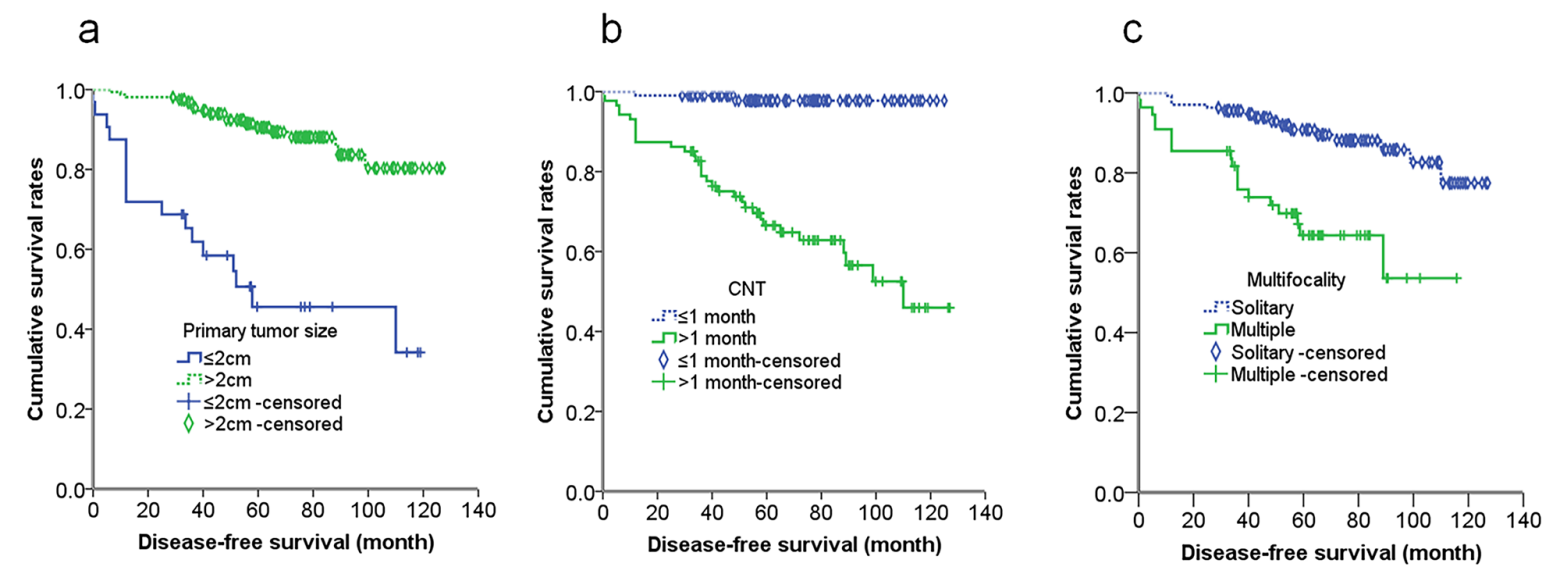
Kaplan–Meier survival curves of MTC patients with primary tumor size (**a**), calcitonin normalization time (**b**) and multifocality (**c**) for DFS. Differences in DFS between groups were significant (*P*<0.05, log-rank test)

**Fig. 7:**
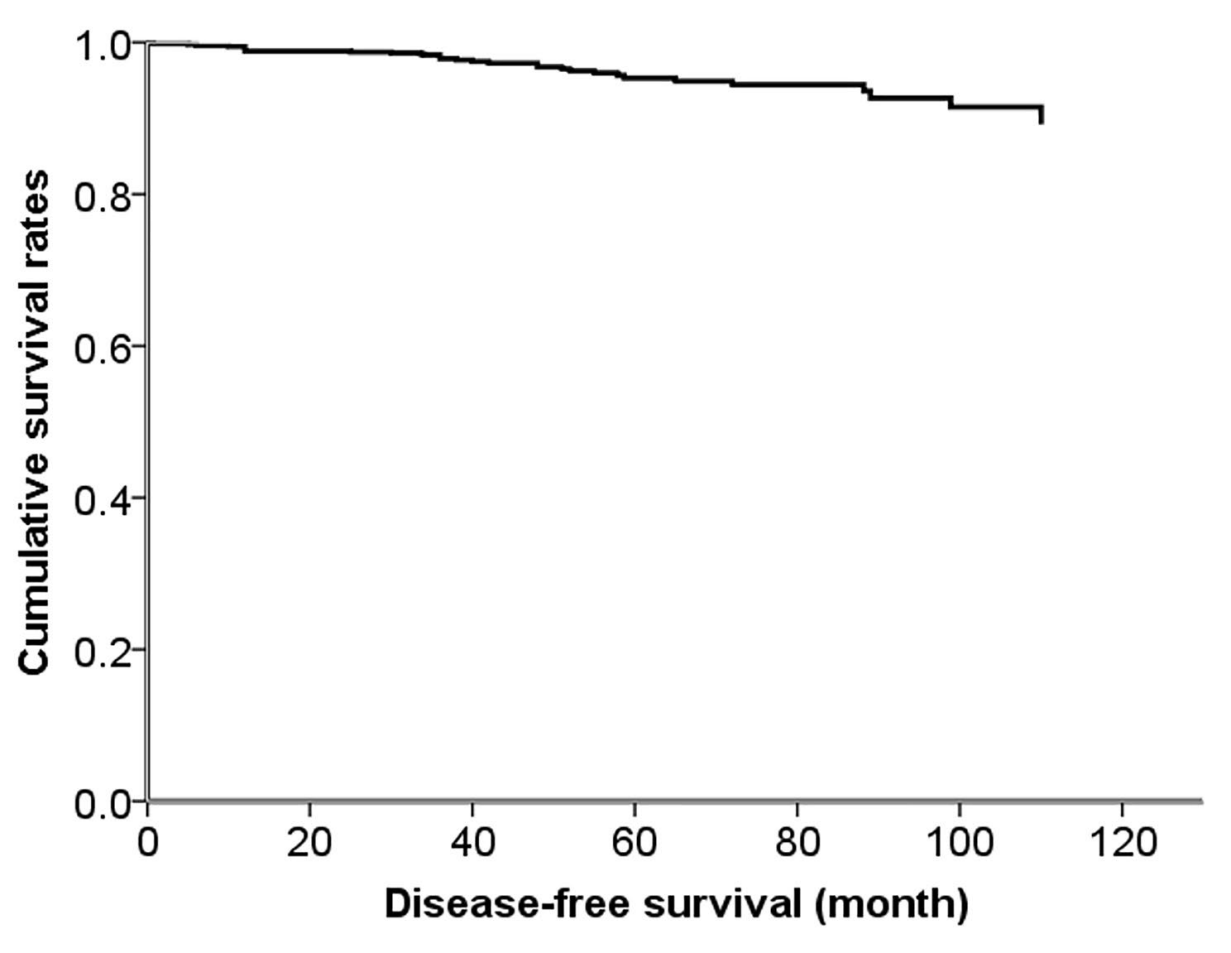
Cumulative survival curve of 190 MTC patients

Male gender, multifocality, extrathyroid invasion, primary tumor size > 2 cm, T3/T4, N1a/N1b, III/IV stage, LNR > 0.23, preoperative serum Ctn level > 1,400 ng/L as well as CNT > 1 month and BPT ≤ 3 month were negative predictors of DFS. In multivariate analysis, primary tumor size > 2 cm (HR, 5.81; 95% CI,2.20-15.38; *P* < 0.01), CNT > 1 month (HR, 5.69; 95% CI, 1.17–27.61; *P* = 0.031).

and multifocality (HR, 3.1;0 95% CI, 1.45–6.65; *P* = 0.004) were independent predictor of DFS. The results were demonstrated in Table 6. The cumulative survival curves with primary tumor size, CNT and multifocality for 190 MTC patients were presented in Fig. 6. The cumulative survival curve of 190 MTC patients was presented in Fig. 7.

**Table 6:** Univariate Kaplan-Meier analysis and multivariate COX analysis for the predictors of disease-free survival (DFS).

Characteristic	Univariate Kaplan-Meier analysis			Multivariate COX analysis		
	¯x ± SD	x^2^	*P*	HR	HR 95.0% CI	*P*
Gender		4.46	0.035			
Female	111.11 ± 3.71					
Male	99.86 ± 5.15			1.10	0.50–2.39	0.816
Age (year)		0.01	0.922			
≤ 50	105.79 ± 3.83					
> 50	106.65 ± 5.30					
Multifocality		17.23	< 0.01			
Solitary	114.07 ± 2.99					
Multiple	81.94 ± 6.31			3.10	1.45–6.65	0.004
Extrathyroid invasion		9.50	0.02			
No	112.25 ± 3.30					
Yes	87.84 ± 6.12			1.24	0.46–3.32	0.672
Primary tumor size (cm)		30.71	< 0.01			
≤ 2	118.07 ± 2.70					
> 2	83.94 ± 6.49			5.81	2.20-15.38	< 0.01
T stage		40.74	< 0.01			
T1/T2	112.71 ± 3.13					
T3/T4	86.13 ± 7.69			0.76	0.25–2.30	0.621
N stage		27.84	< 0.01			
0	123.88 ± 1.76					0.677
N1a	98.90 ± 4.91			0.44	0.05–4.17	0.476
N1b	89.72 ± 5.18			0.39	0.05–3.21	0.384
Clinical stage		25.93	< 0.01			
I/II	125.08 ± 1.31					
III/IV	93.03 ± 4.72			6.31	0.37-107.63	0.203
LNR		37.54	< 0.01			
≤ 0.23	123.82 ± 1.80					
> 0.23	82.53 ± 4.89			2.95	0.64–13.51	0.164
Preoperative serum Ctn(ng/L)		27.96	< 0.01			
≤ 1,400	114.52 ± 3.07					
> 1,400	76.76 ± 6.58			0.95	0.39–2.27	0.900
CNT		37.02	< 0.01			
≤ 1 month	122.91 ± 1.46					
> 1 month	87.62 ± 5.29			5.69	1.17–27.61	0.031
BRT		39.35	< 0.01			
≤ 3 month	66.92 ± 8.85					
> 3 month	114.39 ± 2.77			0.76	0.37–1.58	0.459

*P < 0.05, CNT, calcitonin normalization time; BRT, biochemical persistent/recurrent time; Ctn, calcitonin; DFS, disease-free survival; LNR, lymph node metastasis ratio

## Discussion

Neither effective of radioactive iodine nor the standard chemotherapy or radiotherapy, surgery is recommended treatment for patients with MTC. Despite curative resection of primary tumor, up to 50% of patients do not achieve biochemical cure, as evidenced by persistent elevated Ctn, and 10–25% of patients progressed to structural recurrence ultimately [9–13]. The patients can live asymptomatic with BP for a long period before structural recurrence. Long-term postoperative Ctn normalization, as biochemical cure, is a favorable prognostic factor related to a better outcome, predicting a 10-year survival rate of 97.7% [14, 15]. Any detectable Ctn level value after six months from surgery increased up to 18-fold the risk of persistent disease, independently from tumour size and pre-operative calcitonin levels [16]. High persistent Ctn serum levels represent residual tumor and disease progression. Similar results demonstrated in present study. 95% of (114/120) patients with CN maintained long term biochemical cure and proved no structural recurrence. Patients failed to achieve CN all progressed to BP ultimately. Forty-two percents of patients with BP presented structural recurrence at the last follow up and 65.0% (13/20) patients developed recurrence within 1 month. Short-term Ctn normalization and persistence/recurrence could indicate long term structural recurrence risk. MTC without elevated serum Ctn have been described in literature [17–19]. Gambardella C et al. [19] reviewed 49 patients with definite pathological diagnosis of MTC and normal Ctn. Despite the low or undetectable serum Ctn level, almost half of the tumors presented diffuse or focal positivity for Ctn and CEA immunochemically, and almost all patients were positive for chromogranin A (41/43). Due to the lack of elevation of Ctn and CEA, diagnosis and monitoring of this type of MTC is a challenging.

Most studies chiefly focused on the prognostic role of postoperative Ctn levels for MTC [10, 20–22], little research has been conducted regarding to the temporal dynamics of postoperative Ctn changes and the influencing factors, especially on the BPT. Postoperative Ctn kinetic have been investigated in several surveys and the CNT varies in different literature. Via intro-operative monitoring, serum Ctn eliminated rapidly and declined of 50% by 30 min after curative surgery [2]. Ismailov et al. [3] reported Ctn level could rapid normalized in 2–3 days, slow reduced from 2d -4 weeks or reduced in 7–14 days but subsequently increasing after surgery. Machens et al. [5] reported Ctn levels typically normalized within 1 week postoperative, and took longer depended on more lymph node-positive numbers and higher stratified Ctn levels. For patients destined to achieve a nadir undetectable Ctn as the best response to initial therapy, the Ctn was undetectable in 97% (41/42 patients) by 1 month and 100% by 6 months after curative initial surgery [6]. Consistent with previous observations, CN rapidly achieved within 1 week for 32.5% (39/120) and 1 month for 88.3% (106/120) patients following curative surgery. Except the patients with persistently high Ctn levels, the BP rates of patients gradually increased with time, remarkably differed with CN. The median CNT was 1 month, correspondingly, the median BPT was 6 months. With regard to BPT, most studies have been conducted to identify the important role of Ctn doubling time (Ctn DT) in predicting structural recurrence. Ctn DT has gained increasing interest for its reliability to reflect disease progression and the role of independent predictor of survival [2, 8, 23]. When Ctn DT < 6 months demonstrating the highest, and > 2 years showing the lowest rate of persistent or recurrent disease [24]. Shorter doubling times of serum Ctn correlate with increased mortality [25]. It avoids the biases due to spontaneous variations in the circulating Ctn levels at short-term intervals, however, demands a sufficiently long-term follow up of patients in routine practice for months or even years to calculate it [23, 26, 27], which occasionally hard to apply in practice. Few attentions have payed on exact BPT. We demonstrated dynamic and intuitive time points about CN and BP, providing a new insight for biochemical marker surveillance.

The prognostic roles of CNT and BPT were studied. CTN and BPT were both related to DFS. CNT > 1 month independently correlated with shorter DFS, but BPT was not, which was firstly reported. Similar to previous report, serum Ctn nadirs to undetectable levels within 1 month of curative surgery in MTC suggested low risk of structural disease [6]. We speculated that the difference might due to the short half-life of Ctn, which decreases rapidly after radical surgery of MTC. However, when few residual lesions left after surgery, the time from biochemical recurrence to structural recurrence was longer. Tumor burden, including tumor size and multifocality, affected survival. A study including 1,237 MTC patients from Surveillance, Epidemiology, and End Results (SEER) data displayed tumor size, age, metastasis status, and LNR were selected as independent predictors of overall survival (OS) and cancer-specific survival [28]. Tumor size greater than 2 cm (HR, 2.83; 95% CI, 1.08–7.44 for > 2 to 4 cm and HR, 2.89; 95% CI, 1.09–7.71 for > 4 cm) was proved to be Independent risk factor for disease-specific mortality [29]. Similar results were revealed in other studies [20, 30]. With respect to multifocality, literature only took the largest tumor into consideration, resulting underestimation of tumor volume, generally. Every single thyroid malignancy might contribute to disease development. Machens et al [31] proved tumor multifocality was an independent risk factor of lymph node metastasis on top of primary tumor size when the diameter of the largest primary tumor is the same. Further, prevalence of multifocality was statistically significant increased in advanced T stage and N stag in sporadic MTC [32]. Crucially, multifocality (HR: 8.466, 95% CI: 1.286–55.716, *P* = 0.026) was demonstrated independently correlated with MTC prognosis, and almost all patients with structural persistent disease had multifocal tumors [33].

Moreover, risk factors attributing to the turning point of Ctn dynamic changes remains elusive. A previous study reported that the length of time to Ctn normalization was dependent on both nodal disease burden and preoperative Ctn levels [5]. Ctn levels typically normalize within 1 week; and within a fortnight in those with node-positive MTC and preoperative Ctn levels of 500.1–1000 pg/ml. With node-positive MTC and preoperative Ctn levels exceeding 1000 pg/ml, and with more than ten nodal metastases, Ctn normalization takes longer. Similar results demonstrated in present study. All factors related to CN and BP were all predictors for longer CNT and shorter BPT. After adjusted in multivariate analysis, LNR > 0.23 and male gender were independent factors for CN and BP, and independent predictors for CNT. LNR > 0.23 as well as preoperative serum Ctn > 1,400 ng/L were independent predictors for BPT. Male gender has been reported in previous studies to be related to loco-reginal recurrence/persistence and distant metastases, worse OS [21], overall mortality [20], disease-specific mortality [29], and risk of lateral cervical lymph node metastasis [34]. In our data, male gender was independently relate to CN, CNT and BP. LNR, quantifies the number of lymph node metastases and accurately reflects the extent of surgery, has been reported independently associating with biochemical cure [35, 36], progressive disease [37] and as a predictors of overall survival [28, 38, 39]. Little research has reported the correlation between LNR and CNT, BPT. We demonstrated that LNR > 0.23 was significantly correlated with structural recurrence, and was a independent predictor of prolonged CNT and shortened BPT. As Machens et al. [5] speculated that Ctn-rich lymphatic fluid in node-positive patients needs to be processed through the lymphatic system into the systemic circulation, which takes longer than simple systemic elimination of Ctn in node-negative patients. Our findings strengthen previous conclusions regarding the influence of LNR on patients’ biochemical prognosis.

We further compared clinical and pathological factors related to shorter CNT and longer BPT in subgroup analysis. Advanced N stage was related to CNT ≤ 1 month, larger primary tumor size and higher preoperative serum Ctn was related to BPT > 3 month (all *P* < 0.05). Secreted by C cells, preoperative Ctn correlates with tumor burden, including tumor size [40], lymph node involvement [15, 27], recurrence and metastases [30, 41]. More importantly, preoperative Ctn was identified independently correlated to postoperative Ctn normalization [40]. Intriguingly, the predictive value of preoperative Ctn for BPT is stronger than that for CNT, proving to be an independent predictor for BPT.

As a retrospective study, limitations can’t be neglected. Firstly, other biomarkers, such as CEA, were not taken into consideration since few patients data was available and no dynamic follow up. Secondly, Ctn was tested routinely at 1month intervals post-operation resulting that the exact time of CN and BP can’t be identified. Finally, the patients in our study underwent preoperative vocal cord motility through laryngoscopy, whereas transcutaneous laryngeal ultrasonography was reported as a reliable, non-invasive and inexpensive preoperative method in the evaluation of vocal cords motility [42]. Nevertheless, the dynamic time course of Ctn and risk factors contributing to CNT and BPT in present study, remains valid.

## Conclusions

We conducted a thorough analysis based on a large cohort to evaluate the time kinetics and prognosis roles of CN or BP after Surgery for MTC. In conclusion, early changes of Ctn after surgery can predict the risk of long-term biochemical cure and survival. The tumor burden and Ctn level at the time of initial treatment are important for biochemical prognosis. LNR plays a crucial role in CN and BP, and has been identified as an independent predictor of CNT and BPT. Longer CNT and lager tumor burden indicate shortened DFS. We suggest that early time kinetic of postoperative Ctn can be a useful tool to plan intensity of follow-up.

## Data Availability

No datasets were generated or analysed during the current study.

## Abbreviations

MTC: medullary thyroid cancer
Ctn: calcitonin
CN: calcitonin normalization
BP: biochemical recurrence
LNR: lymph node metastasis ratio
CNT: calcitonin normalization time
BPT: biochemical persistent/recurrent time
DFS: disease-free survival
OS: overall survival
SD: standard deviation
HR: hazard ratio
OR: Odd ratio
CI: confidence interval
